# The dropper stress characteristics under different moving load speeds for a high-speed railway

**DOI:** 10.1016/j.heliyon.2024.e24145

**Published:** 2024-01-05

**Authors:** Fan He, Xinxin Shen, Dandan Guo, Liming Chen, Like Pan

**Affiliations:** a School of Science, Beijing University of Civil Engineering and Architecture, Beijing, 100044, China; b Standards & Metrology Research Institute, China Academy of Railway Sciences Corporation Limited, Beijing, 100081, China

**Keywords:** Vibration, Stress, Moving load, Dropper, Finite difference method

## Abstract

Dropper failure seriously threatens the operation safety of a high-speed railway. In this work, for a simple chain suspension catenary, one span with five droppers is performed to establish a model and thus the effects of the moving load speed on dropper stress are investigated. First, the partial differential vibration equation of dropper is obtained through the mechanical analysis and converted into the finite difference equation. Then, we consider contact line as a beam element to obtain its motion equation. Furthermore, the boundary and initial conditions of five droppers are determined. Finally, the stresses of five droppers are numerically calculated and the effects of the moving load speed on dropper stress are investigated by writing a MATLAB code. The results suggest that the dropper location significantly affects its stress. Compared with other droppers, droppers II and IV have much more severe vibration amplitudes. Different moving load speeds could cause different stress change of each dropper. With the increasing speed, dropper experiences longer bending compression stage and the bending amplitude increases. The impact of the moving load speed on dropper stress is significant.

## 1 Introduction

In a catenary system, dropper withstands vibration and mechanical fatigue, which directly affects driving safety of high-speed railways. In order to ensure the economic and efficient operation of an electrified train, it is of great significance to investigate the factors that affect running safety and stability, including the dropper stress characteristics.

Park built a catenary model using a finite element method [1]. Kim considered the elastic deformations of catenary and established a model to analyze the influences of pre relaxation and tension on the characteristics of catenary based on a finite element method [2]. Other researchers [[3], [4], [5]] investigated pantograph-catenary coupling simulation systems to study the mechanical characteristics. Pombo performed the influences of aerodynamic forces on multi pantograph-catenary coupling [6]. Cho proposed a method of combining a finite element model with numerical time integration to solve the dynamic problem of pantograph overhead contact line [7]. Song constructed a pantograph-catenary model and studied the catenary fluctuation characteristics. The results indicate that the increase of catenary tension makes the catenary fluctuation reflection coefficient decrease [8]. Liu combined the fracture morphology analysis experiment with a finite element simulation analysis of the interface pressing force and concluded that a stress concentration is caused by the reduction of the local copper wire section and the excessive pressing force is the main reason of dropper fracture [9]. Most researchers have focused on mathematical model construction and pantograph-catenary coupling analysis [[10], [11], [12]].

Compared with the dynamics of pantographs, few researches are conducted on the components of catenary. As an important device, dropper has a significant effect on ensuring the safe operation. However, due to the difficulty of collecting related data, there are few studies on the dropper stress characteristics. The mechanical characteristics of catenary system have been more concerned, such as wave propagation, material, and so on [[13], [14], [15], [16], [17]]. However, the detailed mechanical understandings of dropper under different moving load speeds are still absent. This work aims to investigate the effects of the moving load speed on dropper stress. With the continuous improvement of train speed, the railway operation environment is more complex and changeable. The transmission capacity of catenary facilities is more stringent. The failure caused by the broken dropper is increasing gradually. Therefore, the study on the dropper stress characteristics is urgent.

## 2 Vibration equation of dropper

### 2.1 Mechanical analysis for dropper

**Fig. 1:**
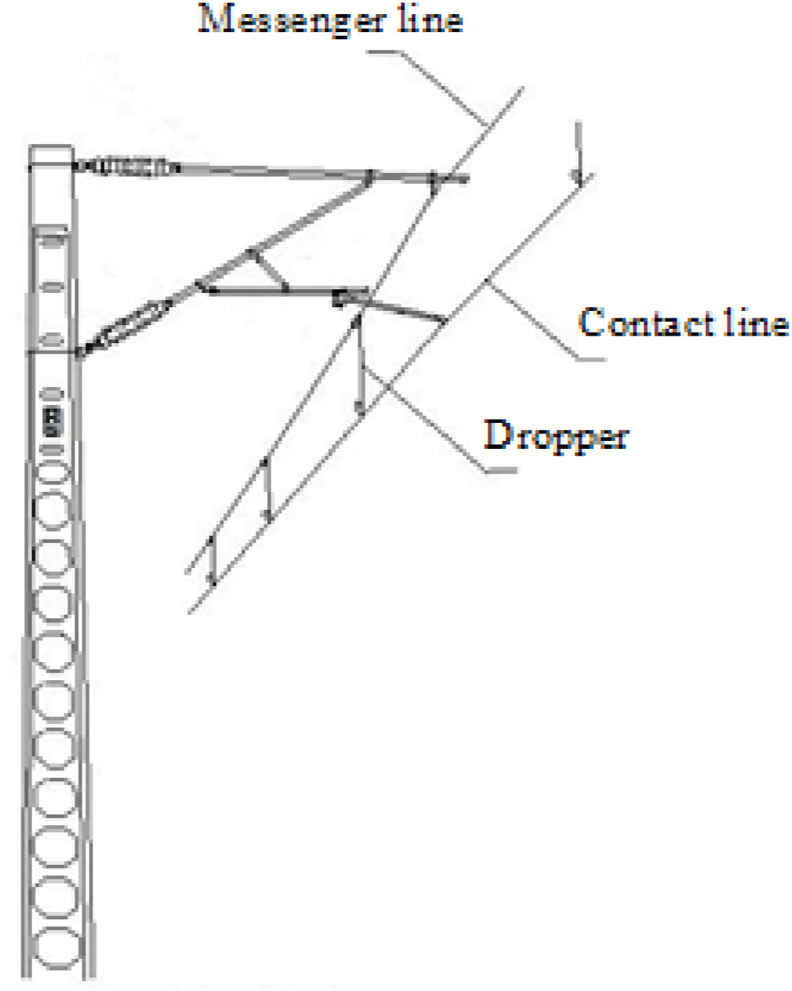
The catenary system.

Fig. 1 shows the catenary system. When a train passes, dropper will vibrate regularly under the action of the pantograph's lifting force. The bending of dropper during vibration process will greatly affect its fatigue life. The dropper stress has been changing during the repeated bending, which is the key reason of the dropper fracture.

**Fig. 2:**
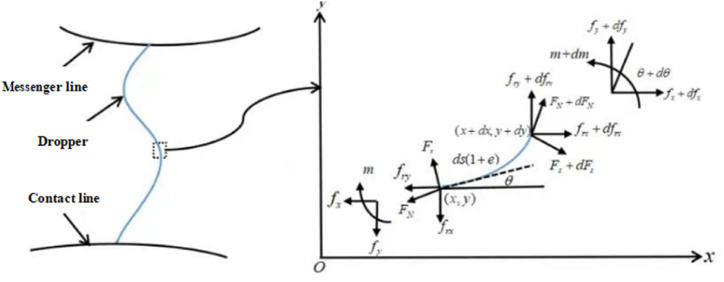
Mechanical analysis diagram of dropper.

Fig. 2 describes mechanical analysis of dropper. Thus, the stress change of dropper could be studied on the basis.

For computational simplicity and convenience, we give the dimensionless variables as follows:

x‾=xLd,y‾=yLd,s‾=sLd,v‾x=vxgLd,v‾y=vygLd,f‾x=fxEdAd,f‾y=fxEdAd,m‾=mEdAdLd,u=ρdgLdEd,t‾=tLd/g,λ2=EdAdLd2EdId,c‾d=cdL2EdAdLdg Through the mechanical analysis, the equations could be written as:

(1) $$\left\{ \begin{array}{l} {\bar{f}}_{x}\;\cos\;\theta +{\bar{f}}_{y}\;\sin\;\theta =\sqrt{{\left(\frac{\partial \bar{x}}{\partial \bar{s}}\right)}^{2}+{\left(\frac{\partial \bar{y}}{\partial \bar{s}}\right)}^{2}}-1\\ \lambda^{2}\bar{m}=\frac{\partial \theta }{\partial \bar{s}}\\ {\bar{f}}_{x}\;\sin\;\theta -{\bar{f}}_{y}\;\cos\;\theta =\frac{\partial \bar{m}}{\partial \bar{s}}\\ \frac{\partial {\bar{f}}_{x}}{\partial \bar{s}}-{\bar{c}}_{d}\frac{\partial \bar{x}}{\partial \bar{t}}=\mu \frac{\partial^{2}\bar{x}}{\partial^{2}\bar{y}}\\ \frac{\partial {\bar{f}}_{y}}{\partial \bar{s}}-{\bar{c}}_{d}\frac{\partial \bar{y}}{\partial \bar{t}}=\mu \frac{\partial^{2}\bar{y}}{\partial t^{2}}+\mu \\ \frac{d\bar{y}}{d\bar{x}}=\tan\;\theta \end{array}\right.$$

Further details of the equation set (1) are shown in Appendix A. The equation set (1) contains $\bar{x},\bar{y},\theta ,\bar{f_{x}},\bar{f_{y}},\bar{m}\text{,}$ 6 dimensionless unknowns, which could be obtained by combining boundary and initial conditions. The calculation of the dropper stress could be performed as:

(2) $$\sigma =\frac{F_{N}}{A_{d}}+\frac{m}{w_{z}}$$

In Eq. (2), *F*_N_ is the axial tensile force, and *m* is the bending moment. They could be obtained by solving the equation set (1). *A*_*d*_ is the cross section area of dropper, and *w*_z_ is its bending section modulus.

### 2.2 The parameters of dropper

Dropper is made by twisting multiple copper wires and its bending stiffness could be obtained by the calculation formula of the stranded wire deduced by Costello [18]. The bending stiffness of dropper in this paper is obtained based on the Costello model. The parameters are shown in Table 1.

**Table 1:** Parameters of dropper.

Cross section area /m^2^	Density /kg·m^−3^	Elastic modulus /Pa	Bending section modulus /m^3^	Bending stiffness /Pa·m^4^	Damping coefficient
1.29 × 10^−5^	8.9 × 10^3^	83.29 × 10^9^	‘6.7663 × 10^−9^	2.53	10

### 2.3 Numerical method

We write a MATLAB code to solve the equation set (1) using a finite difference method. The following difference format is used.

$$\frac{\partial \bar{x}}{\partial \bar{s}}=\frac{{\bar{x}}_{i+1}-{\bar{x}}_{i}}{\Delta \bar{s}}$$

$$\frac{\partial \bar{y}}{\partial \bar{s}}=\frac{{\bar{y}}_{i+1}-{\bar{y}}_{i}}{\Delta \bar{s}}$$

$$\frac{\partial \theta }{\partial \bar{s}}=\frac{\theta_{i+1}-\theta_{i}}{\Delta \bar{s}}$$

$$\frac{\partial \bar{m}}{\partial \bar{s}}=\frac{{\bar{m}}_{i+1}-{\bar{m}}_{i}}{\Delta \bar{s}}$$

$$\frac{\partial {\bar{f}}_{x}}{\partial \bar{s}}=\frac{{\bar{f_{x}}}_{i+1}-{\bar{f_{x}}}_{i}}{\Delta \bar{s}}$$

$$\frac{\partial {\bar{f}}_{y}}{\partial \bar{s}}=\frac{{\bar{f_{y}}}_{i+1}-{\bar{f_{y}}}_{i}}{\Delta \bar{s}}$$

$$\frac{d\bar{y}}{d\bar{x}}=\frac{{\bar{y}}_{i+1}-{\bar{y}}_{i}}{{\bar{x}}_{i+1}-{\bar{x}}_{i}}$$

$$\frac{\partial^{2}\bar{x}}{\partial {\bar{t}}^{2}}=\frac{{\bar{x}}_{j+2}-2{\bar{x}}_{j+1}+{\bar{x}}_{j}}{\Delta {\bar{t}}^{2}}$$

$$\frac{\partial^{2}\bar{y}}{\partial {\bar{t}}^{2}}=\frac{{\bar{y}}_{j+2}-2{\bar{y}}_{j+1}+{\bar{y}}_{j}}{\Delta {\bar{t}}^{2}}$$

Here, $\Delta \bar{s}=\frac{1}{n}$ is the dimensionless element length, *n* is the discrete element number. Each element has an equal length. $\bar{x_{i}},\bar{y_{i}}\left(i=0,1,2\cdot \cdot \cdot \cdot \cdot \cdot n\right)$ are the dimensionless coordinates of the *i*th element, $\Delta \bar{t}$ is the time step, and $\bar{t_{j}}=j\Delta \bar{t}\left(j=0,1,2\cdot \cdot \cdot \cdot \cdot \cdot \right)$ is the discretization time. We select *n* = 32 and $\Delta \bar{t}=7.5\times 10^{-10}$ according to the result error and the calculation time.

## 3 Derivation and solution of vibration equation of contact line

### 3.1 Catenary model

**Fig. 3:**
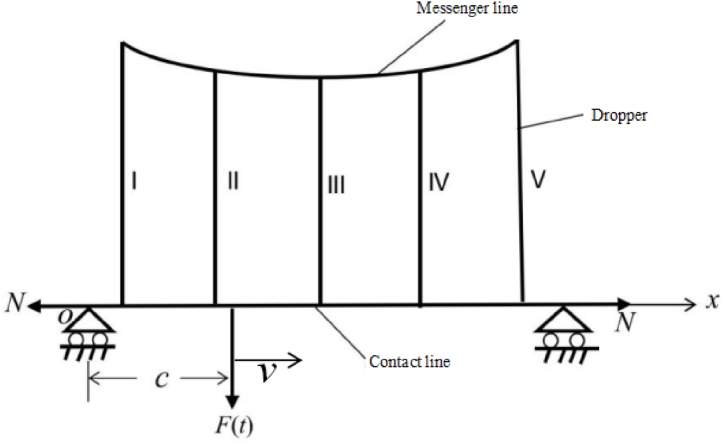
The model of the simple catenary system.

Through the mechanical analysis, we obtain the vibration equation of dropper. To get its solution, the boundary and initial conditions must be involved. The determination of the conditions requires the establishment of a catenary model. It is shown in Fig. 3.

In Fig. 3, F(t) represents the force of pantograph on catenary. *N* is the tension of contact line. Under actual working conditions, the pantograph-catenary interaction is very complicated and data collection is very difficult. Sinusoidal force is often used in the experiment for simulation, the force amplitude is often selected as 100∼400 N, and the frequency is often selected as 1∼10Hz [18]. In this work, the force amplitude and frequency is respectively selected as 100 N and 1Hz. *c* is the load location away from the origin. The distance between every two dropper is 10 m and droppers I and V have 1.6 m lengths. Droppers II and IV have 1.295 m lengths. Dropper III has a 1.2547 m length.

### 3.2 Vibration equation of contact line

**Fig. 4:**
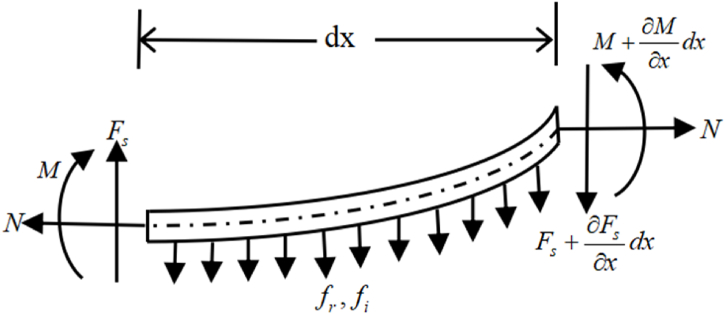
Mechanical analysis diagram of micro-segment contact line considering tension.

To obtain the vibration equation of contact line, we perform the mechanical analysis of contact line, as shown in Fig. 4.Where, *dx* is the micro-segment length, $f_{r}$ is the damping force when it moves vertically, $f_{i}$ is the inertial force, $F_{s}$ is the shear force, *M* denotes the bending moment.

The details of the vibration equation are shown in Appendix B. Under different moving load speeds, the vibration response of each dropper is different. To investigate the effects of moving load speed on dropper stress, we select three cases of 250, 300 and 350 km/h in this paper. Only the first five orders are calculated because the influence of higher orders could be ignored. Thus, we write the following equation of contact line.

(3) $$y\left(x,t\right)=\sum_{i=1}^{5}[\frac{-\frac{F}{2}Y\left(x\right)\cos\left(\omega t+\frac{i\pi \left(c+vt\right)}{l}\right)}{\frac{EIi^{4}\pi^{4}}{2l^{3}}+\frac{Ni^{2}\pi^{2}}{2l}+\frac{{\left[\frac{Cl}{2}\left(\omega +\frac{i\pi v}{l}\right)\right]}^{2}}{\frac{EIi^{4}\pi^{4}}{2l^{3}}+\frac{Ni^{2}\pi^{2}}{2l}-\frac{\rho Al}{2}{\left(\omega +\frac{i\pi v}{l}\right)}^{2}}-\frac{\rho Al}{2}{\left(\omega +\frac{i\pi v}{l}\right)}^{2}}+\frac{\frac{F}{2}Y\left(x\right)\cos\left(\omega -\frac{i\pi \left(c+vt\right)}{l}\right)}{\frac{EIi^{4}\pi^{4}}{2l^{3}}+\frac{Ni^{2}\pi^{2}}{2l}+\frac{{\left[\frac{Cl}{2}\left(\omega -\frac{i\pi v}{l}\right)\right]}^{2}}{\frac{EIi^{4}\pi^{4}}{2l^{3}}+\frac{Ni^{2}\pi^{2}}{2l}-\frac{\rho Al}{2}{\left(\omega -\frac{i\pi v}{l}\right)}^{2}}-\frac{\rho Al}{2}{\left(\omega -\frac{i\pi v}{l}\right)}^{2}}+\frac{-\frac{FCl}{4}\left(\omega +\frac{i\pi v}{l}\right)Y\left(x\right)\sin\left(\omega +\frac{i\pi \left(c+vt\right)}{l}\right)}{\left[\frac{EIi^{4}\pi^{4}}{2l^{3}}+\frac{Ni^{2}\pi^{2}}{2l}+\frac{{\left[\frac{Cl}{2}\left(\omega +\frac{i\pi v}{l}\right)\right]}^{2}}{\frac{EIi^{4}\pi^{4}}{2l^{3}}+\frac{Ni^{2}\pi^{2}}{2l}-\frac{\rho Al}{2}{\left(\omega -\frac{i\pi v}{l}\right)}^{2}}-\frac{\rho Al}{2}{\left(\omega +\frac{i\pi v}{l}\right)}^{2}\right]\cdot \left[\frac{EIi^{4}\pi^{4}}{2l^{3}}+\frac{Ni^{2}\pi^{2}}{2l}-\frac{\rho Al}{2}{\left(\omega +\frac{i\pi v}{l}\right)}^{2}\right]}+\frac{\frac{FCl}{4}\left(\omega -\frac{i\pi v}{l}\right)Y\left(x\right)\sin\left(\omega -\frac{i\pi \left(c+vt\right)}{l}\right)}{\left[\frac{EIi^{4}\pi^{4}}{2l^{3}}+\frac{Ni^{2}\pi^{2}}{2l}+\frac{{\left[\frac{Cl}{2}\left(\omega -\frac{i\pi v}{l}\right)\right]}^{2}}{\frac{EIi^{4}\pi^{4}}{2l^{3}}+\frac{Ni^{2}\pi^{2}}{2l}-\frac{\rho Al}{2}{\left(\omega -\frac{i\pi v}{l}\right)}^{2}}-\frac{\rho Al}{2}{\left(\omega -\frac{i\pi v}{l}\right)}^{2}\right]\cdot \left[\frac{EIi^{4}\pi^{4}}{2l^{3}}+\frac{Ni^{2}\pi^{2}}{2l}-\frac{\rho Al}{2}{\left(\omega -\frac{i\pi v}{l}\right)}^{2}\right]}]$$

Substituting x=5,15,25,35,45,v=250,300,350 km/h into Eq. (3), we could get the vertical displacements of five droppers under different speeds. The bending stiffness of contact line is 233.92 Pa m^4^ according to the Costello model and its elastic modulus is 118.49 × 10^9^ Pa. The cross section area of contact line is 1.58 × 10^−4^ m^2^ and its density is 8.9 × 10^3^ kg m^−3^. In addition, we set *N* = 30 kN, *F* = 100 N, ω=2π, *C* = 1.

## 4 3 boundary and initial conditions

**Fig. 5:**
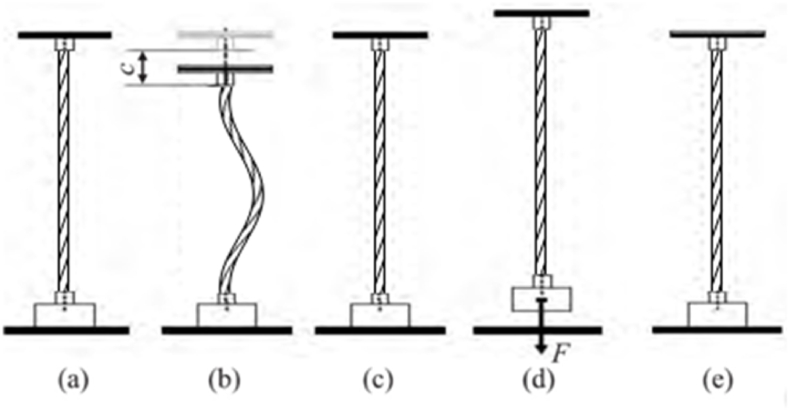
The dropper behaviour under loading.

Fig. 5 is a schematic diagram of dropper behaviour under loading. Fig. 5(a), (b), (c), (d) and (e) represent different states under actual conditions. The detailed descriptions could be found in the previous work [19].

According to the above analysis, regardless of the moving load speed and the dropper location, the boundary and initial conditions of five droppers could be uniformly written as

(4) $$\left\{ \begin{array}{l} \bar{x}\left(\bar{s},\bar{t}\right)=\frac{5+10\left(i-1\right)}{L_{i}}\\ \bar{y}\left(\bar{s},\bar{t}\right)=\bar{s} \end{array}\right.⤢\;when\;\bar{t}=0\text{.}$$

(5) $$\left\{ \begin{array}{l} \bar{x}\left(\bar{s},\bar{t}\right)=\frac{5+10\left(i-1\right)}{L_{i}}\\ \bar{y}\left(\bar{s},\bar{t}\right)=\sum_{i=1}^{5}[\frac{A_{i}}{L_{i}}\cos\left(2\pi \sqrt{\frac{L_{i}}{g}}\cdot \bar{t}+\frac{i\pi \left(c+v\sqrt{\frac{L_{i}}{g}}\cdot \bar{t}\right)}{l}\right)\\ +\frac{B_{i}}{L_{i}}\cos\left(2\pi \sqrt{\frac{L_{i}}{g}}\cdot \bar{t}-\frac{i\pi \left(c+v\sqrt{\frac{L_{i}}{g}}\cdot \bar{t}\right)}{l}\right)\\ +\frac{C_{i}}{L_{i}}\sin\left(2\pi \sqrt{\frac{L_{i}}{g}}\cdot \bar{t}+\frac{i\pi \left(c+v\sqrt{\frac{L_{i}}{g}}\cdot \bar{t}\right)}{l}\right)\\ +\frac{D_{i}}{L_{i}}\sin\left(2\pi \sqrt{\frac{L_{i}}{g}}\cdot \bar{t}-\frac{i\pi \left(c+v\sqrt{\frac{L_{i}}{g}}\cdot \bar{t}\right)}{l}\right)] \end{array}\right.⤢⤢when\;\bar{s}=0\text{.}$$

(6) $$\left\{ \begin{array}{l} \bar{x}\left(\bar{s},\bar{t}\right)=\frac{5+10\left(i-1\right)}{L_{i}}\\ \bar{y}\left(\bar{s},\bar{t}\right)\leq 1 \end{array}\right.⤢⤢when\;\bar{s}=1\text{.}$$

In Eqs. (4), (5), (6), the subscript *i* could be set from 1 to 5, which represents the *i*th dropper. $L_{i}$ is different dropper length. $A_{i},B_{i},C_{i}D_{i}$ denote the amplitude of the vertical displacement.

## 5 Results

### 5.1 Stresses of five droppers under 250 km/h moving load

**Fig. 6:**
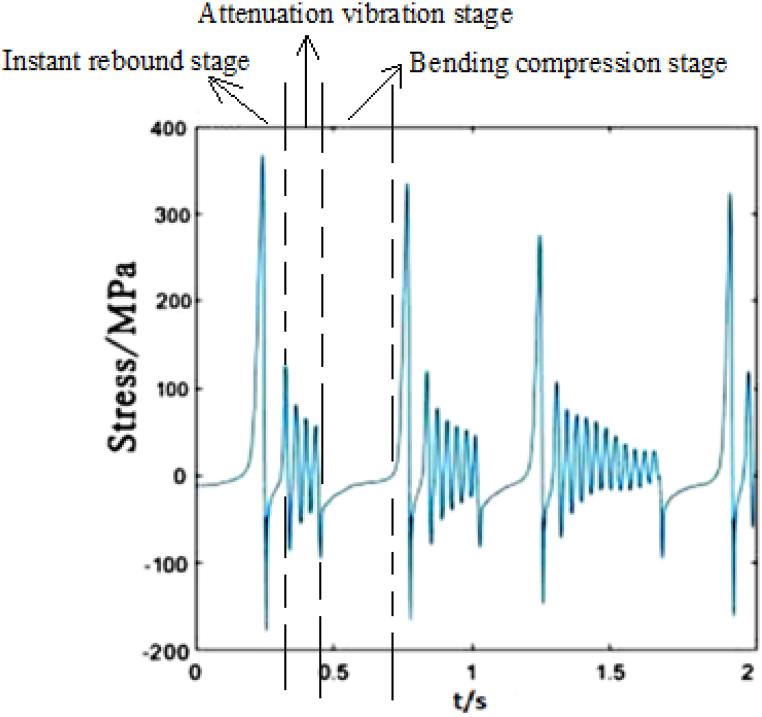
The time-dependent stress of dropper I.

Fig. 6 plots the stress change of dropper I under 250 km/h moving load. The contact line vibrates due to the moving load acting on it, which makes the dropper vibrate and its stress change. During the stress change process, the peak stresses reach very quickly. The stress variation experiences three stages, that is, instant rebound, attenuation vibration and bending compression stages.

**Fig. 7:**
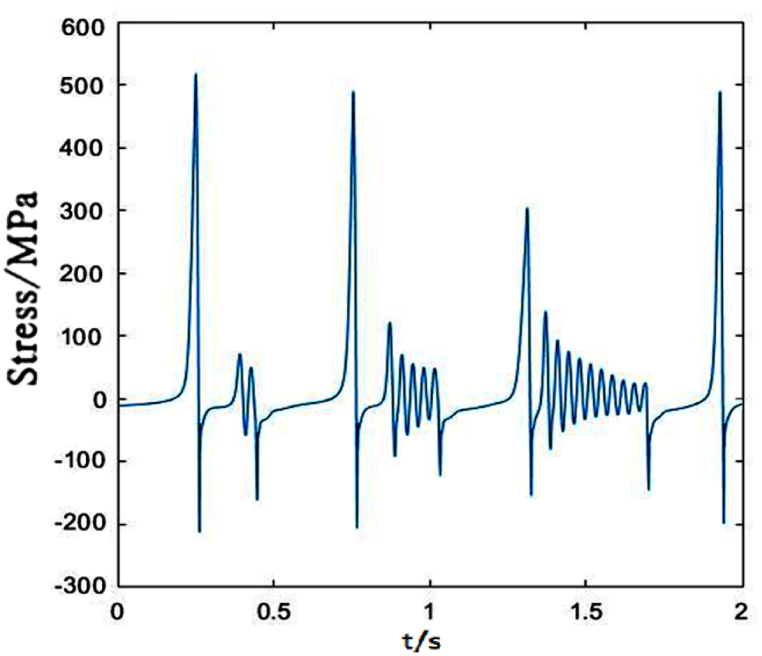
The time-dependent stress of dropper II.

Due to different locations of droppers II and I, the stress changes of them are also different. The greatest differences are the maximum tensile and compressive stresses. The load acting on the catenary makes the dropper vibrate and the oscillation propagates in wave from the left to right. When the load acts on a certain part of the contact line, there will be energy loss during wave propagation. Therefore, the increasing distance away from the load location makes the vibration amplitude decrease gradually. However, in this paper, because the load sustains to act on the contact line, dropper II withstands the higher maximum tensile and compressive stresses than dropper I, as shown in Fig. 7.

**Fig. 8:**
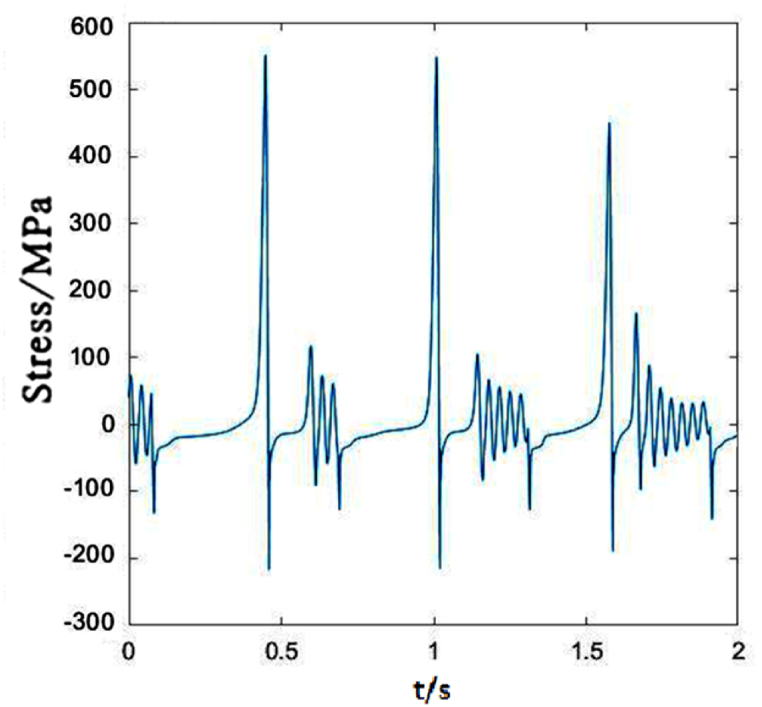
The time-dependent stress of dropper III.

Fig. 8 depicts the time-dependent stress of dropper III. It is not obvious different from those of droppers I and II. In a short time, the tensile and compressive stresses reach maximum. However, the time to reach the maximum stresses is slightly different, and its vibration degree is also more intense than that of dropper I.

**Fig. 9:**
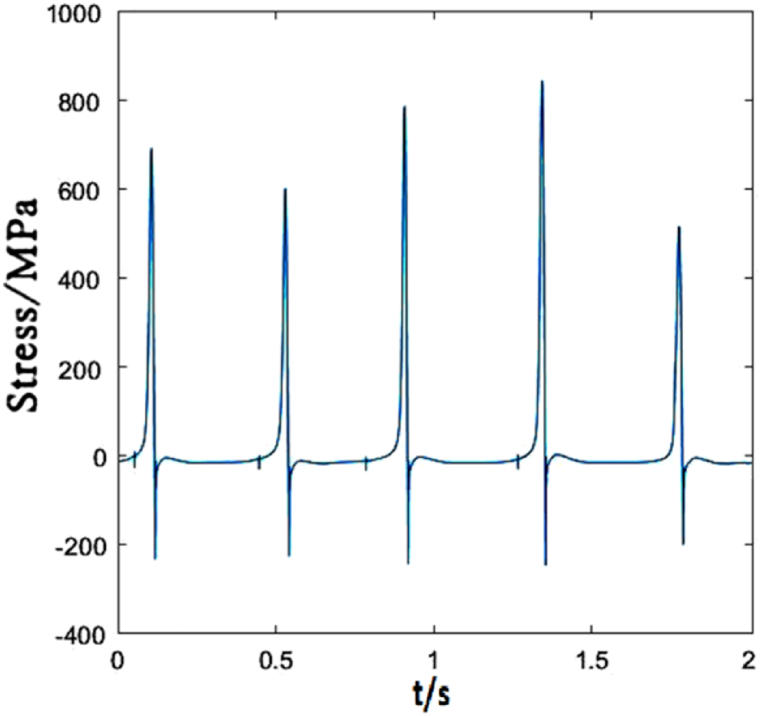
The time-dependent stress of dropper IV.

The time-dependent stress of dropper IV is shown in Fig. 9. The difference between dropper IV and droppers I, II and III is that there is almost no attenuation vibration stage. There is a followed large bending stage when the tensile and compressive stresses reach maximum in a short time.

**Fig. 10:**
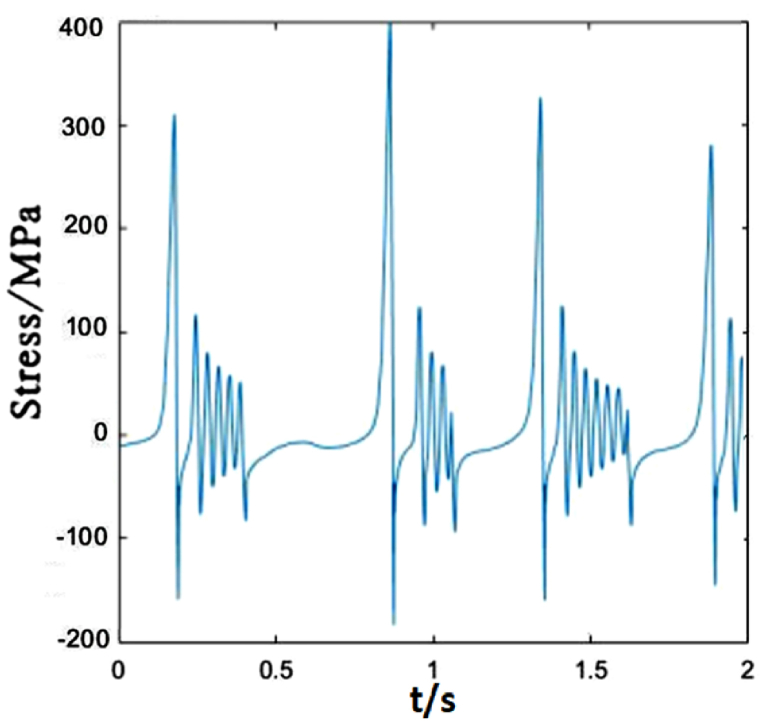
The time-dependent stress of dropper V.

The time-dependent stress of dropper V is identical to those of the first three droppers, as shown in Fig. 10. They all go through three stages. However, due to the different locations in the catenary, the maximums of the tensile and compressive stresses are different.

### 5.2 Stresses of five droppers under 300 km/h moving load

**Fig. 11:**
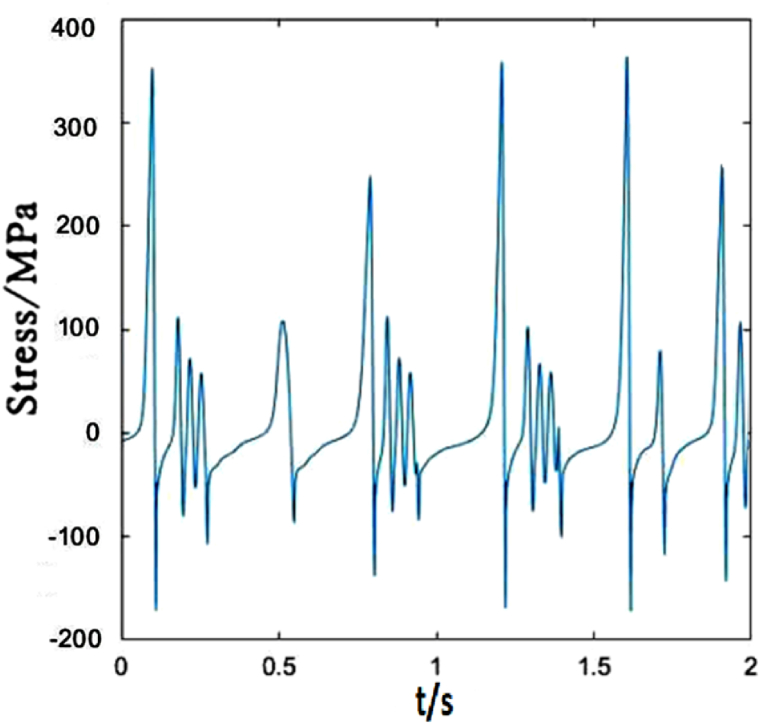
The time-dependent stress of dropper I.

Fig. 11 shows the stress change of dropper I under 300 km/h moving load. It could be observed that the time-dependent stress of the dropper is the same as that under 250 km/h moving load, and the maximum changes of the tensile and compressive stresses are slight.

**Fig. 12:**
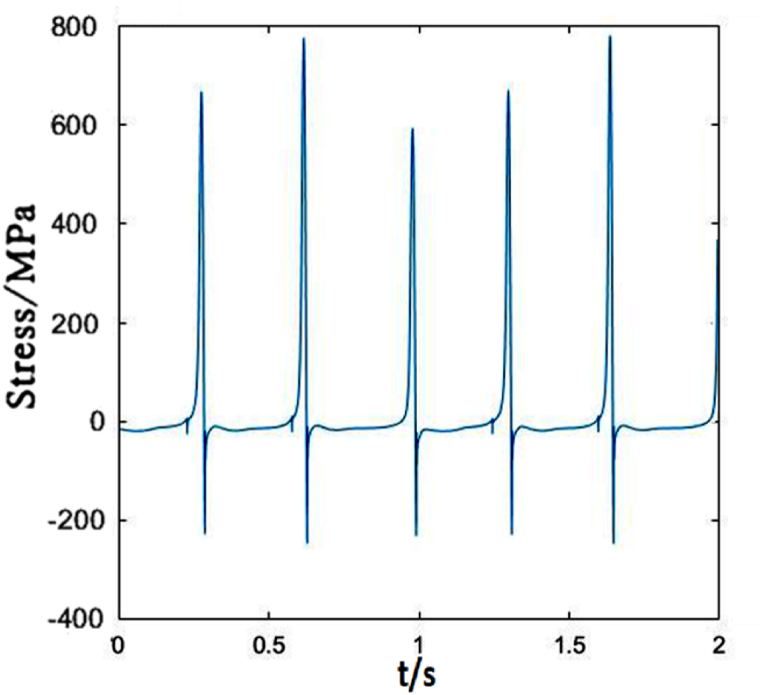
The time-dependent stress of dropper II.

The time-dependent stress of dropper II is shown in Fig. 12. It is observed that the stress change is more sensitive to the variable speed than that of dropper I. When the speed increases from 250 to 300 km/h, the maximums of the tensile and compressive stresses of dropper I almost do not change. However, the maximum of the tensile stress of dropper II is 1.5 times than that under 250 km/h moving load.

**Fig. 13:**
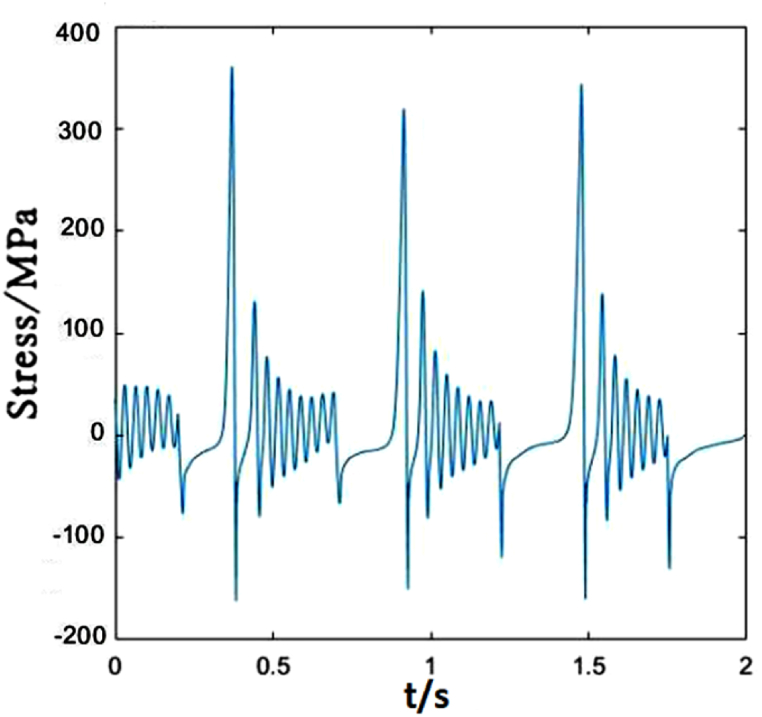
The time-dependent stress of dropper III.

The time-dependent stress of dropper III is the same as that of dropper I. However, its vibration amplitude is weaker than that of dropper II, as shown in Fig. 13.

**Fig. 14:**
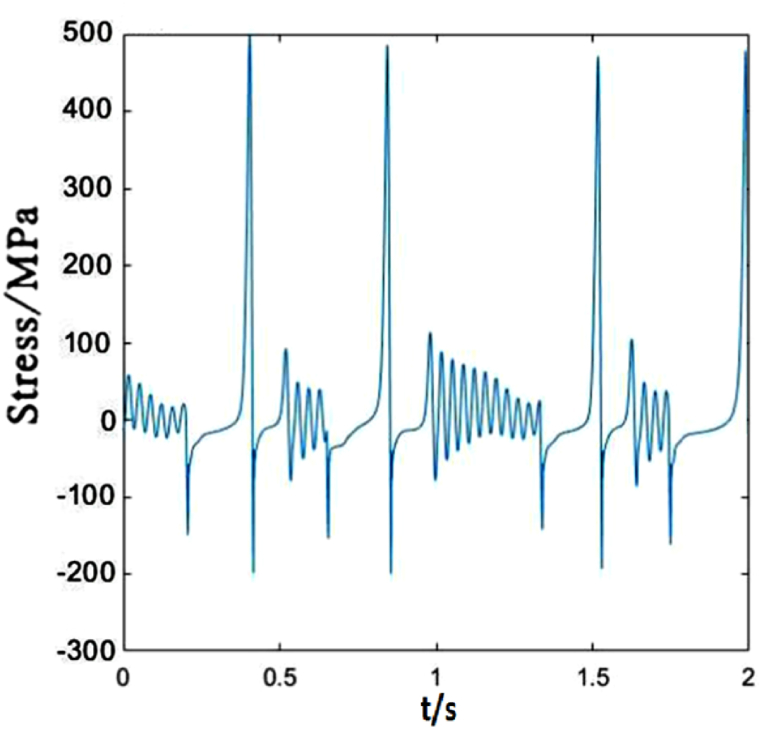
The time-dependent stress of dropper IV.

Fig. 14 represents dropper IV is the same as sensitive to the speed change as dropper II. The maximum tension and compression stresses of the dropper markedly change with the increasing speed.

**Fig. 15:**
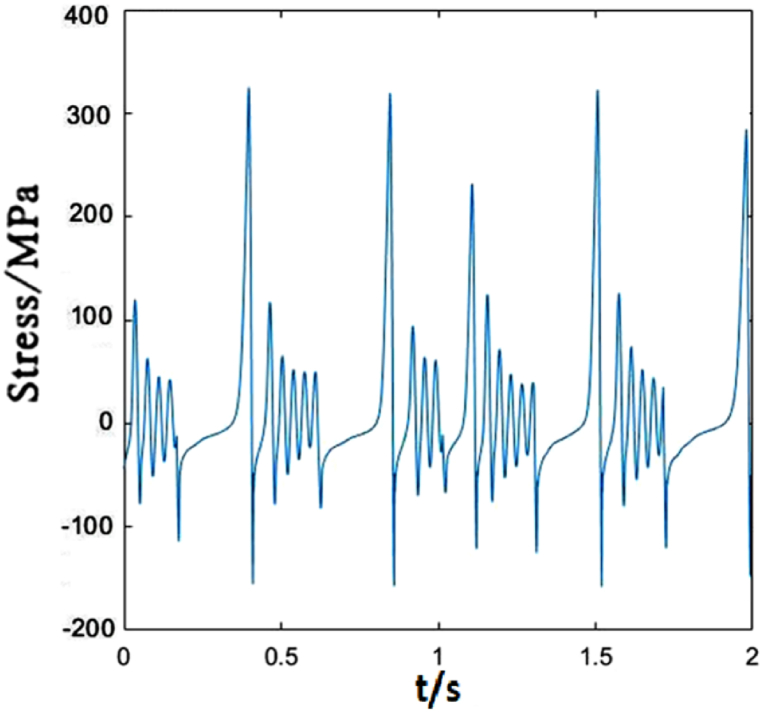
The time-dependent stress of dropper V.

It could be seen from Fig. 15 that the time-dependent stress of dropper V undergoes three stages no matter under 250 or 300 km/h moving loads, and the maximums of the tensile and compressive stresses are not obviously affected by the speed.

### 5.3 Stresses of five droppers under 350 km/h moving load

**Fig. 16:**
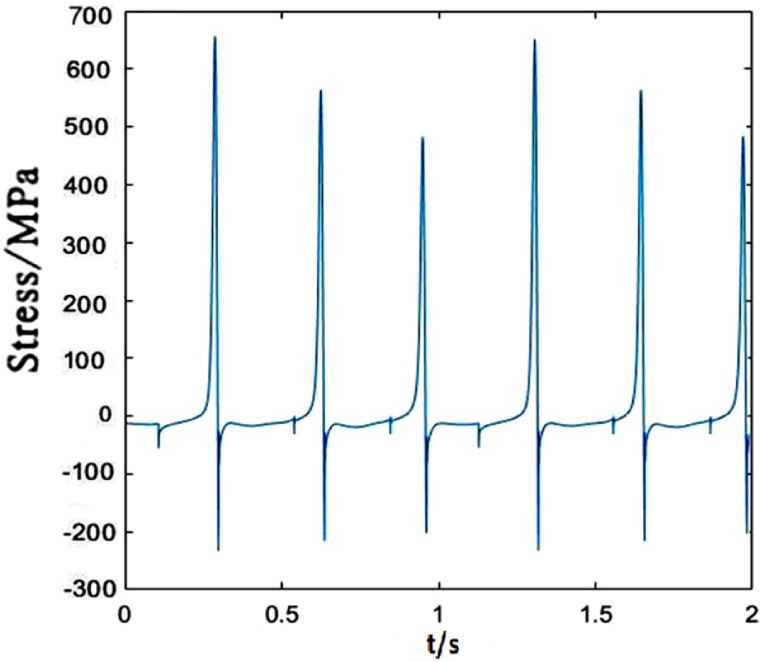
The time-dependent stress of dropper I.

When the moving load speed is 350 km/h, the dropper I stress changes obviously and the vibration amplitude increases with the increasing speed. There is almost no attenuation vibration stage, as shown in Fig. 16. The maximum of the tensile stress is approximately 1.74 times than that under 250 km/h moving load.

**Fig. 17:**
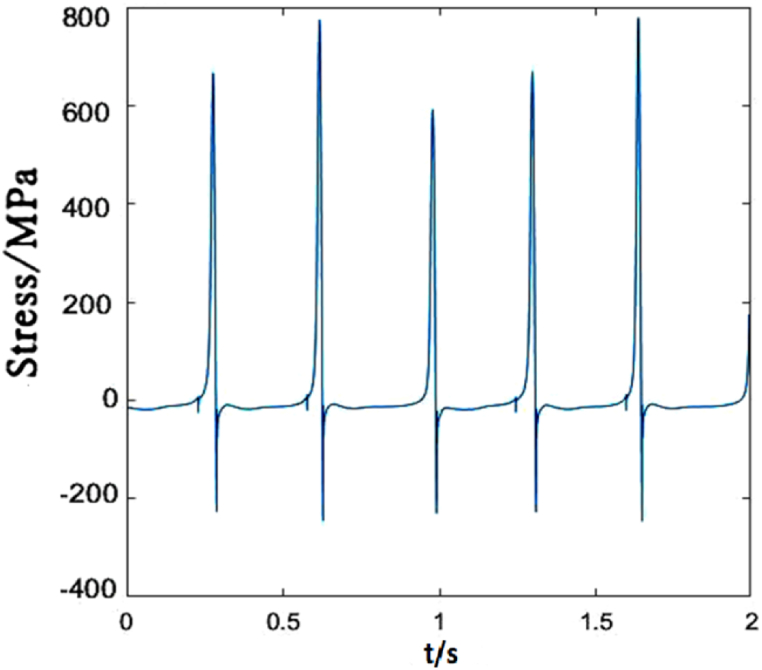
The time-dependent stress of dropper II.

Compared with dropper I, the time-dependent stress of dropper II does not also experience attenuation vibration stage. Fig. 17 shows that dropper II significantly endures the higher maximum of the tensile stress than dropper I.

**Fig. 18:**
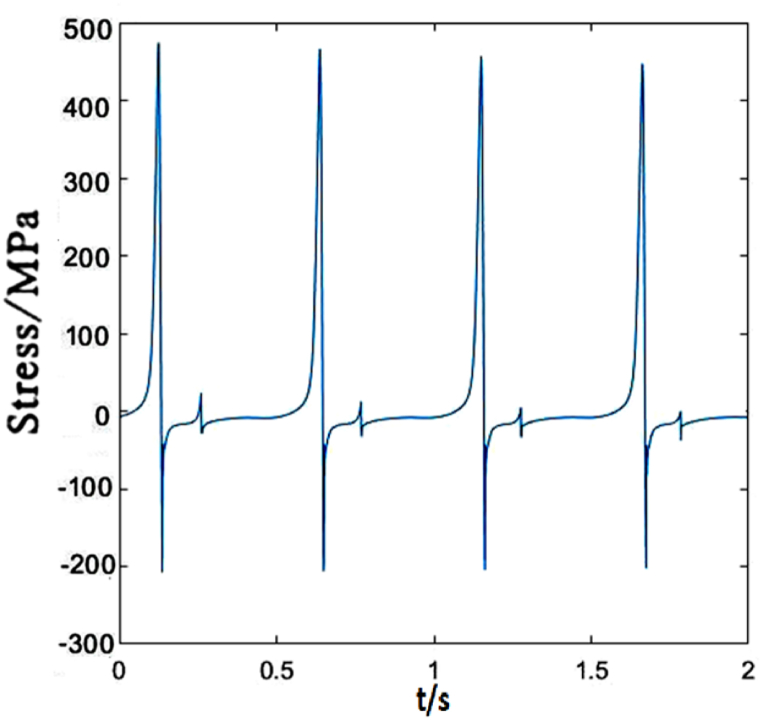
The time-dependent stress of dropper III.

Dropper III has the weaker vibration amplitude than dropper II when the moving load speed is 350 km/h, which is the same as those under 250 and 300 km/h moving loads, as shown in Fig. 18.

**Fig. 19:**
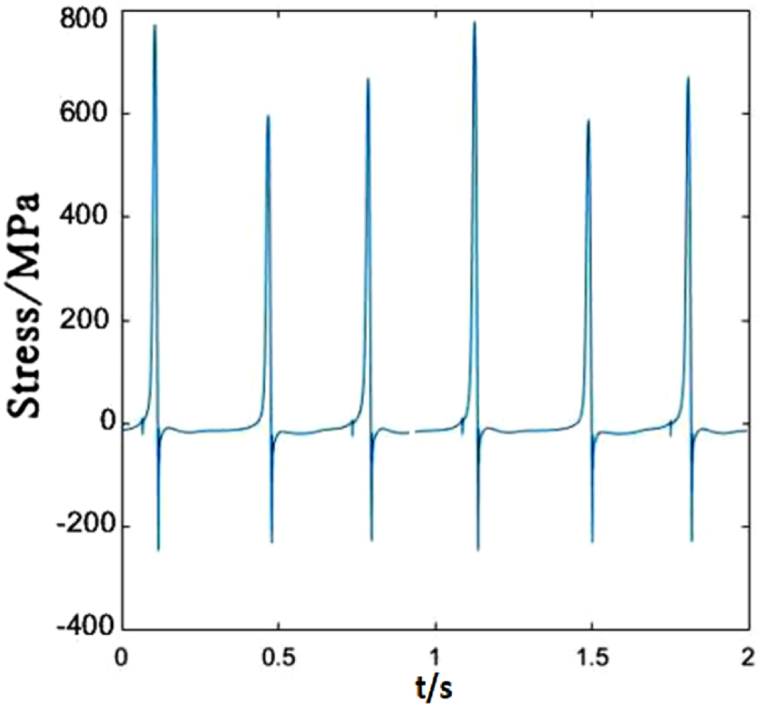
The time-dependent stress of dropper IV.

Fig. 19 describes that the time-dependent stress of dropper IV is the same as that of dropper II. It is also more sensitive to the variable speed than those of other droppers.

**Fig. 20:**
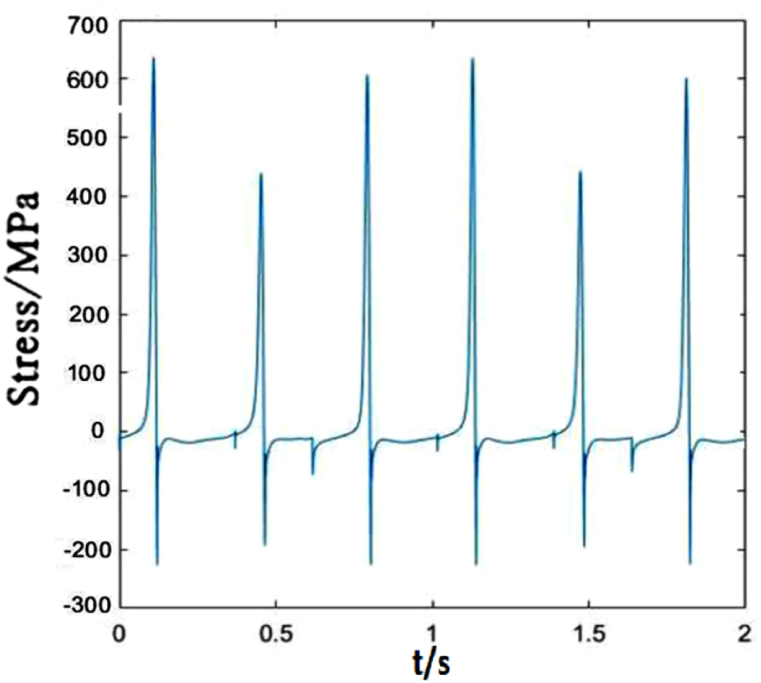
The time-dependent stress of dropper V.

Fig. 20 plots that the stress change of dropper V is more severe than those under 250 and 300 km/h moving loads. When the moving load speed attains to 350 km/h, it is found that the stress vibrations of all droppers experience two stages and a large bending stage occurs during the stress change process.

## 6 Discussion

The maximum tensile and compressive stress values under different moving load speeds are shown in Table 2. No matter where the dropper is located in the catenary, each dropper will vibrate and its stress will change because the vibration propagates in the form of wave.

**Table 2:** Maximum stresses of five droppers.

Dropper No.	250 km/h		300 km/h		350 km/h	
	Maximum tensile stress (MPa)	Maximum compressive stress (MPa)	Maximum tensile stress (MPa)	Maximum compressive stress (MPa)	Maximum tensile stress (MPa)	Maximum compressive stress (MPa)
Dropper I	380	190	380	180	660	240
Dropper II	520	210	780	240	780	220
Dropper III	580	220	360	160	480	210
Dropper IV	820	220	500	200	780	240
Dropper V	400	190	320	160	650	230

Table 2 shows the maximum stresses of five droppers under different moving load speeds. It could be found that the dropper location has a very important impact on its stress. It is found that no matter how fast the moving load is, at the same speed, the stress values of droppers II or IV are highest. Thus, it is more easily to fracture for droppers II and IV. According to Yu's experimental statistics, Table 3 shows that droppers II and IV fracture more frequently [20]. Hence, our results are found to be in excellent agreement with the experimental results.

**Table 3:** Statistics of dropper fracture for a high-speed railway.

Dropper No.	Quantity	Proportion
Dropper I	98	15.3 %
Dropper II	122	21 %
Dropper III	103	17.7 %
Dropper IV	156	26.8 %
Dropper V	111	19.2 %

According to the stress variations of droppers under 250, 300 and 350 km/h moving loads, it could be found that the stress is affected by the moving load speed. When the speed is 250 or 300 km/h, most of droppers will experience three stages during their stress change process. Namely, instant rebound, attenuation vibration and bending compression stages. The differences are that the duration of the three stages is different and the maximum stresses are different. Under 350 km/h moving load, there are only two stages for a span of droppers except attenuation vibration stage. Furthermore, the duration of bending compression stage is longer than that under 250 and 300 km/h moving loads. It is found that the longer the attenuation time, the weaker the bending amplitude, and the longer the bending compression stage, the stronger the bending amplitude. Under 350 km/h moving load, the spent time in the attenuation vibration stage is much shorter than that under 250 and 300 km/h moving loads. The reason is that the droppers under 350 km/h moving load are subjected to a greater impact force, more severe vibration and bending than those under other moving load speeds.

## 7 Conclusion

In this paper, dropper stress calculations under 250, 300, and 350 km/h moving loads are performed. The vibration equation of dropper is obtained through the mechanical analysis. At the same time, a beam element is used to treat the contact line. Thus, the motion equation is obtained and the boundary and initial conditions of five droppers are determined. The time-dependent stresses of five droppers under different moving load speeds are calculated. Through the analysis of the dropper stress, the following conclusions could be obtained.

(1) The bending amplitude of dropper increases significantly with the increasing moving load speed. The higher the speed, the longer the bending process lasts and more intense the bending amplitude becomes.
(2) The load location markedly affects dropper stress. Under the same moving load speed, the stresses of droppers II and IV are the most sensitive to the variable speed, and their maximum stresses are also highest. Therefore, controlling the train speed could effectively make the stress amplitude and maximum tensile stress of dropper decrease. Hence, the working reliability of dropper could be improved.

## Data availability statement

Data included in article/supplementary material/referenced in article.

## Additional information

No additional information is available for this paper.

## CRediT authorship contribution statement

**Fan He:** Writing - review & editing, Writing - original draft, Methodology, Conceptualization. **Xinxin Shen:** Formal analysis. **Dandan Guo:** Investigation. **Liming Chen:** Validation. **Like Pan:** Validation.

## Declaration of competing interest

The authors declare that they have no known competing financial interests or personal relationships that could have appeared to influence the work reported in this paper.

## Appendix A

The combination of axial tension and shear force of micro-element section could be written as follows.

(A-1) $$F_{N}=f_{x}\;\cos\;\theta +f_{y}\;\sin\;\theta$$

(A-2) $$F_{s}=f_{x}\;\sin\;\theta -f_{y}\;\cos\;\theta$$

Where, the axial tensile and shear forces are denoted by $F_{N}$ and $F_{S}$, θ is the angle between the positive *x*-direction and the micro-element segment, *f*_*x*_ and *f*_*y*_ are the partial *x*-direction and *y*-direction forces, respectively. According to Hooke's law, stress is proportional to strain.

(A-3) $$\delta_{d}=E_{d}e$$

Where, $\delta_{d}$ is the axial stress of dropper, $E_{d}$ is its elastic modulus, and *e* is the axial strain of the micro-element section. According to the definition of stress

(A-4) $$\delta_{d}=\frac{F_{N}}{A_{d}}$$

Thus, the expression of the axial strain is the following.

(A-5) $$e=\frac{F_{N}}{E_{d}A_{d}}=\frac{f_{x}\;\cos\;\theta +f_{y}\;\sin\;\theta }{E_{d}A_{d}}$$

According to the definition of strain,

(A-6) $$e=\frac{ds^{\prime }-ds}{ds}=\sqrt{{\left(\frac{\partial x\left(s,t\right)}{\partial s}\right)}^{2}+{\left(\frac{\partial y\left(s,t\right)}{\partial s}\right)}^{2}}-1$$

Where, ds and d $s^{\prime }$ are the original and deformed length of dropper. At the same time, the relationship between the arc length and the micro-element section location *x*, *y* is as follows.

(A-7) $$\frac{\partial x\left(s,t\right)}{\partial s}=\left(1+e\right)\cos\;\theta$$

(A-8) $$\frac{\partial y\left(s,t\right)}{\partial x}=\left(1+e\right)\sin\;\theta$$

By the definition of curvature

(A-9) $$k=\frac{\partial \theta }{\partial s}=\frac{m}{E_{d}I_{d}}$$

Where, *k* is the micro-element section curvature, and the exact differential equation for the deflection curve could be obtained by using mechanics of materials. The bending moment and stiffness of dropper are represented by *m* and $E_{d}I_{d}$.

According to the relationship between shear force and bending moment, we could write the following equation.

(A-10) $$\frac{\partial m}{\partial s}F_{s}=f_{x}\;\sin\;\theta -f_{y}\;\cos\;\theta$$

The micro-element motion equation of dropper is

(A-11) $$\frac{\partial f_{x}}{\partial s}-c_{d}v_{x}=\frac{\partial f_{x}}{\partial s}-c_{d}\frac{\partial x}{\partial t}=\rho_{d}A_{d}\frac{\partial^{2}x}{\partial t^{2}}$$

(A-12) $$\frac{\partial f_{y}}{\partial s}-c_{d}v_{y}=\frac{\partial f_{y}}{\partial s}-c_{d}\frac{\partial y}{\partial t}=\rho_{d}A_{d}\frac{\partial^{2}y}{\partial t^{2}}+\rho_{d}A_{d}g$$

Where, $c_{d}$ is the damping coefficient of dropper, $\rho_{d}$ is its density.

In addition, there is the following equation.

(A-13) $$\frac{dy}{dx}=\tan\;\theta$$

## Appendix B

According to the balance principle of force, the sum of the projection forces in the vertical direction is zero, that is,

(B-1) $$F_{s}-\left(F_{s}+\frac{\partial F_{s}}{\partial x}dx\right)-f_{r}dx-f_{i}dx=0$$

Where $f_{i}dx$ is the laterally distributed inertial force, and it could be calculated as

(B-2) $$f_{i}dx=\rho Adx\frac{\partial^{2}y\left(x,t\right)}{\partial t^{2}}$$

Where *y* (*x*,*t*) is the vertical position of contact line.

Bringing Eq. (B-2) into Eq. (B-1), it could be simplified as

(B-3) $$\frac{\partial F_{s}}{\partial x}=-\rho A\frac{\partial^{2}y\left(x,t\right)}{\partial t^{2}}-f_{r}$$

When the axial force of contact line is studied, the effects of tension and shear forces are mainly considered. The following balance equation could be listed.

(B-4) $$M+F_{s}dx+N\frac{\partial y\left(x,t\right)}{\partial x}dx-f_{r}\frac{dx}{2}-pAdx\frac{\partial^{2}y\left(x,t\right)}{\partial t^{2}}\frac{dx}{2}-\left(M+\frac{\partial M}{\partial x}dx\right)=0$$

Introducing the bending stiffness *EI*, the following relationship could be established from the mechanics of materials.

(B-5) $$M=EI\frac{\partial^{2}y\left(x,t\right)}{\partial x^{2}}$$

Thus,

(B-6) $$F_{s}=-N\frac{\partial y\left(x,t\right)}{\partial x}+\frac{\partial }{\partial x}\left[EI\frac{\partial y^{2}\left(x,t\right)}{\partial x^{2}}\right]$$

Substituting Eq. (B-6) into Eq. (B-3), it could be obtained

(B-7) $$\frac{\partial }{\partial x}\left[-N\frac{\partial y\left(x,t\right)}{\partial x}+\frac{\partial }{\partial x}\left(EI\frac{\partial y^{2}\left(x,t\right)}{\partial x^{2}}\right)\right]=-\rho A\frac{\partial^{2}y\left(x,t\right)}{\partial t^{2}}-f_{r}$$

Where $f_{r}=C\frac{\partial y\left(x,t\right)}{\partial t}$, *C* is the damping coefficient of contact line. Thus,

(B-8) $$EI\frac{\partial^{4}y\left(x,t\right)}{\partial x^{4}}-N\frac{\partial^{2}y\left(x,t\right)}{\partial x^{2}}+\rho A\frac{\partial^{2}y\left(x,t\right)}{\partial t^{2}}+C\frac{\partial y\left(x,t\right)}{\partial t}=0$$

Where, A is the cross section area of contact line and ρ is its density.

Suppose the main vibration:

(B-9) y(x,t)=Y(x)sin(ωt+φ)

Bringing Eq. (B-9) into Eq. (B-8), we could get

(B-10) $$EIY^{⁗}\left(x\right)\sin\left(\omega t+\varphi \right)-NY^{\prime \prime }\left(x\right)\sin\left(\omega t+\varphi \right)+C\omega Y\left(x\right)\cos\left(\omega t+\varphi \right)+\rho A\omega^{2}Y\left(x\right)\sin\left(\omega t+\varphi \right)=0$$

The four characteristic values of the equation could be obtained through calculation.

(B-11) $$\lambda_{12}=\pm \sqrt{\frac{N}{2EI}+\sqrt{{\left(\frac{N}{2EI}\right)}^{2}+\frac{\rho A}{EI}\omega^{2}+\frac{C\;\cos\left(\omega t+\varphi \right)}{EI\;\sin\left(\omega t+\varphi \right)}\omega }}=\pm a\left(t\right)$$

(B-12) $$\lambda_{34}=\pm i\sqrt{-\frac{N}{2EI}+\sqrt{{\left(\frac{N}{2EI}\right)}^{2}+\frac{\rho A}{EI}\omega^{2}+\frac{C\;\cos\left(\omega t+\varphi \right)}{EI\;\sin\left(\omega t+\varphi \right)}\omega }}=\pm ib\left(t\right)$$

The general solution of Eq. (B-10) could be obtained as:

(B-13) $$Y\left(x\right)=A_{1}\;\sinh\;a\left(t\right)x+A_{2}\;\cosh\;a\left(t\right)x+A_{3}\;\sin\;b\left(t\right)x+A_{4}\;\cos\;b\left(t\right)x$$

We treat contact line as a beam element. Due to supports, the bending moment and deflection at both ends are zero.

(B-14) $$\left\{ \begin{array}{l} Y\left(0\right)=0\\ Y\left(l\right)=0\\ Y^{\prime \prime }\left(0\right)=0\\ Y^{\prime \prime }\left(l\right)=0 \end{array}\right.$$

Thus, it could be obtained as:

(B-15) A1=A2=A=40

The frequency equation is

(B-16) $$\sin\;b_{i}\left(t\right)l=0$$

The solution is

(B-17) $$b_{i}\left(t\right)l=i\pi \left(i=1,2,3,\cdots \cdots \right)$$

From the frequency equation, the modal function could be obtained as:

(B-18) $$Y_{i}\left(x\right)=\sin\frac{i\pi }{l}x\left(i=1,2,3\cdots \cdots \right)$$

Under the action of a travelling concentrated force at a speed *v*, the forced vibration equation is

(B-19) $$EI\frac{y^{4}\left(x,t\right)}{\partial x^{4}}-N\frac{\partial^{2}y\left(x,t\right)}{\partial x^{2}}+C\frac{\partial y\left(x,t\right)}{\partial t}+\rho A\frac{\partial^{2}y\left(x,t\right)}{\partial t^{2}}=F\left(t\right)\delta \left(x-\left(c+vt\right)\right)$$

Suppose the solution of Eq. (B-19)

(B-20) $$y\left(x,t\right)=\sum_{i=1}^{\infty }Y_{i}\left(x\right)q_{i}\left(t\right)$$

Substituting Eq. (B-20) into Eq. (B-19), we could get

(B-21) $$\sum_{i=1}^{\infty }\left[EIq_{i}\left(t\right)Y_{i}^{⁗}\left(x\right)-NY_{i}^{\prime \prime }\left(x\right)q_{i}\left(t\right)+CY\left(x\right)q_{i}^{\prime }\left(t\right)+\rho AY_{i}\left(x\right)q_{i}^{\prime \prime }\left(t\right)\right]=F\left(t\right)\delta \left(x-\left(c+vt\right)\right)$$

After multiplying both sides of Eq. (B-21) by $Y_{j}\left(x\right)$ and integrating x along the length, the following equation could be obtained.

(B-22) $$\begin{array}{l} EIq_{i}\left(t\right)\int_{0}^{l}\sum_{i=1}^{\infty }Y_{i}^{⁗}\left(x\right)Y_{j}\left(x\right)dx-Nq_{i}\left(t\right)\int_{0}^{l}\sum_{i=1}^{\infty }Y_{i}^{\prime \prime }\left(x\right)Y_{j}\left(x\right)dx+\\ Cq_{i}^{\prime \prime }\left(t\right)\int_{0}^{l}\sum_{0}^{l}Y_{i}\left(x\right)Y_{j}\left(x\right)dx\;+\rho Aq_{i}^{\prime \prime }\left(t\right)\int_{0}^{l}\sum_{0}^{l}Y_{i}\left(x\right)Y_{j}\left(x\right)dx=\int_{0}^{l}F\left(t\right)\delta \left(x-\left(c+vt\right)\right)Y_{j}\left(x\right)dx \end{array}$$

The following function properties could be used

(B-23) $${Y_{i}}^{\left(2n\right)}\left(x\right)={\left(-1\right)}^{n}{\left(\frac{i\pi }{l}\right)}^{2n}Y_{i}\left(x\right)$$

(B-24) $$\int_{0}^{l}Y_{i}\left(x\right)Y_{j}\left(x\right)dx=\frac{l}{2}\left(i=j\right)$$

(B-25) $$\int_{0}^{l}Y_{i}\left(x\right)Y_{j}\left(x\right)dx=0\left(i\neq j\right)$$

Eq. (B-22) could be written as:

(B-26) $$\left(\frac{EIi^{4}\pi^{4}}{2l^{3}}+\frac{Ni^{2}\pi^{2}}{2l}\right)q_{i}\left(t\right)+\frac{Cl}{2}q_{i}^{\prime }\left(t\right)+\frac{\rho Al}{2}q_{i}^{\prime \prime }\left(t\right)=F\left(t\right)\sin\frac{i\pi \left(c+vt\right)}{l}$$

Here, we set F(t)=Fsinωt. Thus,

(B-27) $$q_{i}\left(t\right)=r_{1}\;\cos\left(\omega t+\frac{i\pi \left(c+vt\right)}{l}\right)+r_{2}\;\cos\left(\omega t-\frac{i\pi \left(c+vt\right)}{l}\right)+r_{3}\;\sin\left(\omega t+\frac{i\pi \left(c+vt\right)}{l}\right)+r_{4}\;\sin\left(\omega -\frac{i\pi \left(c+vt\right)}{l}\right)$$

$r_{1},r_{2},r_{3},r_{4}$ inhomogeneous linear equations could be written as:

(B-28) $$\left\{ \begin{array}{l} \left(\frac{EIi^{4}\pi^{4}}{2l^{3}}+\frac{Ni^{2}\pi^{2}}{2l}\right)r_{1}+\frac{Cl}{2}r_{3}\left(\omega +\frac{i\pi vt}{l}\right)-\frac{\rho Al}{2}{\left(\omega +\frac{i\pi vt}{l}\right)}^{2}r_{1}=-\frac{F}{2}\\ \left(\frac{EIi^{4}\pi^{4}}{2l^{3}}+\frac{Ni^{2}\pi^{2}}{2l}\right)r_{2}+\frac{Cl}{2}r_{3}\left(\omega +\frac{i\pi vt}{l}\right)-\frac{\rho Al}{2}{\left(\omega -\frac{i\pi vt}{l}\right)}^{2}r_{2}=\frac{F}{2}\\ \left(\frac{EIi^{4}\pi^{4}}{2l^{3}}+\frac{Ni^{2}\pi^{2}}{2l}\right)r_{3}+\frac{Cl}{2}r_{1}\left(\omega +\frac{i\pi vt}{l}\right)-\frac{\rho Al}{2}{\left(\omega +\frac{i\pi vt}{l}\right)}^{2}r_{3}=0\\ \left(\frac{EIi^{4}\pi^{4}}{2l^{3}}+\frac{Ni^{2}\pi^{2}}{2l}\right)r_{4}-\frac{Cl}{2}r_{2}\left(\omega -\frac{i\pi vt}{l}\right)-\frac{\rho Al}{2}{\left(\omega -\frac{i\pi vt}{l}\right)}^{2}r_{4}=0 \end{array}\right.$$

By solving the followings

(B-29) $$\left\{ \begin{array}{l} r_{1}=\frac{-\frac{F}{2}}{\frac{EIi^{4}\pi^{4}}{2l^{3}}+\frac{Ni^{2}\pi^{2}}{2l}+\frac{{\left[\frac{Cl}{2}(\omega +\frac{i\pi v)}{l})\right]}^{2}}{\frac{EIi^{4}\pi^{4}}{2l^{3}}+\frac{Ni^{2}\pi^{2}}{2l}-\frac{\rho Al}{2}{\left(\omega +\frac{i\pi v}{l}\right)}^{2}}-\frac{\rho Al}{2}{\left(\omega +\frac{i\pi vt}{l}\right)}^{2}}\\ r_{2}=\frac{\frac{F}{2}}{\frac{EIi^{4}\pi^{4}}{2l^{3}}+\frac{Ni^{2}\pi^{2}}{2l}+\frac{{\left[\frac{Cl}{2}\left(\omega -\frac{i\pi v}{l}\right)\right]}^{2}}{\frac{EIi^{4}\pi^{4}}{2l^{3}}+\frac{Ni^{2}\pi^{2}}{2l}-\frac{\rho Al}{2}{\left(\omega -\frac{i\pi v}{l}\right)}^{2}}-\frac{\rho Al}{2}{\left(\omega -\frac{i\pi v}{l}\right)}^{2}}\\ r_{3}=\frac{-\frac{FCl}{4}\left(\omega +\frac{i\pi v}{l}\right)}{\left[\frac{EIi^{4}\pi^{4}}{2l^{3}}+\frac{Ni^{2}\pi^{2}}{2l}+\frac{{\left[\frac{Cl}{2}\left(\omega +\frac{i\pi v}{l}\right)\right]}^{2}}{\frac{EIi^{4}\pi^{4}}{2l^{3}}+\frac{Ni^{2}\pi^{2}}{2l}-\frac{\rho Al}{2}{\left(\omega +\frac{i\pi v}{l}\right)}^{2}}-\frac{\rho Al}{2}{\left(\omega +\frac{i\pi v}{l}\right)}^{2}\right]\left[\frac{EIi^{4}\pi^{4}}{2l^{3}}+\frac{Ni^{2}\pi^{2}}{2l}-\frac{\rho Al}{2}{\left(\omega +\frac{i\pi v}{l}\right)}^{2}\right]}\\ r_{4}=\frac{\frac{FCl}{4}\left(\omega -\frac{i\pi v}{l}\right)}{\left[\frac{EIi^{4}\pi^{4}}{2l^{3}}+\frac{Ni^{2}\pi^{2}}{2l}+\frac{{\left[\frac{Cl}{2}\left(\omega -\frac{i\pi v}{l}\right)\right]}^{2}}{\frac{EIi^{4}\pi^{4}}{2l^{3}}+\frac{Ni^{2}\pi^{2}}{2l}-\frac{\rho Al}{2}{\left(\omega -\frac{i\pi v}{l}\right)}^{2}}-\frac{\rho AL}{2}{\left(\omega -\frac{i\pi v}{l}\right)}^{2}\right]\left[\frac{EIi^{4}\pi^{4}}{2l^{3}}+\frac{Ni^{2}\pi^{2}}{2l}-\frac{\rho Al}{2}{\left(\omega -\frac{i\pi v}{l}\right)}^{2}\right]} \end{array}\right.$$

Thus, we get the response of contact line as:

(B-30) $$y\left(x,t\right)=\sum_{i=1}^{n}Y_{i}\left(x\right)q_{i}\left(t\right)=\sum_{i=1}^{\infty }[\frac{-\frac{F}{2}Y_{i}\left(x\right)\cos\left(\omega t+\frac{i\pi \left(c+vt\right)}{l}\right)}{\frac{EIi^{4}\pi^{4}}{2l^{3}}+\frac{Ni^{2}\pi^{2}}{2l}+\frac{{\left[\frac{Cl}{2}\left(\omega +\frac{i\pi v}{l}\right)\right]}^{2}}{\frac{EIi^{4}\pi^{4}}{2l^{3}}+\frac{Ni^{2}\pi^{2}}{2l}-\frac{\rho Al}{2}{\left(\omega +\frac{i\pi v}{l}\right)}^{2}}-\frac{\rho Al}{2}{\left(\omega +\frac{i\pi v}{l}\right)}^{2}}+\frac{\frac{F}{2}Y_{i}\left(x\right)\cos\left(\omega -\frac{i\pi \left(c+vt\right)}{l}\right)}{\frac{EIi^{4}\pi^{4}}{2l^{3}}+\frac{Ni^{2}\pi^{2}}{2l}+\frac{{\left[\frac{Cl}{2}\left(\omega -\frac{i\pi v}{l}\right)\right]}^{2}}{\frac{EIi^{4}\pi^{4}}{2l^{3}}+\frac{Ni^{2}\pi^{2}}{2l}-\frac{\rho Al}{2}{\left(\omega -\frac{i\pi v}{l}\right)}^{2}}-\frac{\rho Al}{2}{\left(\omega -\frac{i\pi v}{l}\right)}^{2}}+\frac{-\frac{FCl}{4}\left(\omega +\frac{i\pi v}{l}\right)Y_{i}\left(x\right)\sin\left(\omega +\frac{i\pi \left(c+vt\right)}{l}\right)}{\left[\frac{EIi^{4}\pi^{4}}{2l^{3}}+\frac{Ni^{2}\pi^{2}}{2l}+\frac{{\left[\frac{Cl}{2}\left(\omega +\frac{i\pi v}{l}\right)\right]}^{2}}{\frac{EIi^{4}\pi^{4}}{2l^{3}}+\frac{Ni^{2}\pi^{2}}{2l}-\frac{\rho Al}{2}{\left(\omega -\frac{i\pi v}{l}\right)}^{2}}-\frac{\rho Al}{2}{\left(\omega +\frac{i\pi v}{l}\right)}^{2}\right]\cdot \left[\frac{EIi^{4}\pi^{4}}{2l^{3}}+\frac{Ni^{2}\pi^{2}}{2l}-\frac{\rho Al}{2}{\left(\omega +\frac{i\pi v}{l}\right)}^{2}\right]}+\frac{\frac{FCl}{4}\left(\omega -\frac{i\pi v}{l}\right)Y_{i}\left(x\right)\sin\left(\omega -\frac{i\pi (c+vt}{l}\right)}{\left[\frac{EIi^{4}\pi^{4}}{2l^{3}}+\frac{Ni^{2}\pi^{2}}{2l}+\frac{{\left[\frac{Cl}{2}\left(\omega -\frac{i\pi v}{l}\right)\right]}^{2}}{\frac{EIi^{4}\pi^{4}}{2l^{3}}+\frac{Ni^{2}\pi^{2}}{2l}-\frac{\rho Al}{2}{\left(\omega -\frac{i\pi v}{l}\right)}^{2}}-\frac{\rho Al}{2}{\left(\omega -\frac{i\pi v}{l}\right)}^{2}\right]\cdot \left[\frac{EIi^{4}\pi^{4}}{2l^{3}}+\frac{Ni^{2}\pi^{2}}{2l}-\frac{\rho Al}{2}{\left(\omega -\frac{i\pi v}{l}\right)}^{2}\right]}]$$
